# In vivo effect of hydroxyethyl starch solution (HES 130/0.4) on different fibrinogen assays

**DOI:** 10.1186/cc13345

**Published:** 2014-03-17

**Authors:** U Schött, DW Winstedt, AH Hillarp

**Affiliations:** 1 Lund University, Lund, Sweden

## Introduction

Previous *in vitro *studies have shown that photometric assays may overestimate fibrinogen levels after hemodilution with HES. The *in vivo *effect of HES on fibrinogen assays was therefore studied.

## Methods

Forty patients with intracranial tumor gave their consent to participate in this ethical approved study. Plasma fibrinogen levels were analyzed with ELISA, two photometric assays (Dade and Multifibren) and one mechanical (Hook). In addition, ROTEM FibTEM- MCF was analyzed.

## Results

**Figure 1:**
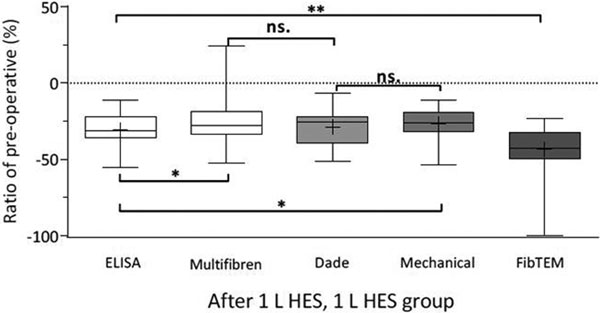
**Relative change of P-fibrinogen compared with preoperative values**.

Twenty-five of the 40 patients received 1 l HES. Mean reduction of hematocrit was 17%. ELISA was lower than Hook and Multifibren. The FibTEM relative decrease of 43% differed significantly from the other assays. See Figure 1.

## Conclusion

After *in vivo *hemodilution with HES, some assays overestimated fibrinogen levels. An overestimation of around 0.3 g/l may lead to undercorrection of fibrinogen in critical bleeding situations. The aggravated response on the FibTEM-MCF may better reflect HES effects on clot structure.

